# Multiple Approaches to Investigate the Transport and Activity-Dependent Release of BDNF and Their Application in Neurogenetic Disorders

**DOI:** 10.1155/2012/203734

**Published:** 2012-06-06

**Authors:** David Hartmann, Jana Drummond, Erik Handberg, Sharday Ewell, Lucas Pozzo-Miller

**Affiliations:** Department of Neurobiology, SHEL 1002, Civitan International Research Center, The University of Alabama at Birmingham, Birmingham, AL 35294-2182, USA

## Abstract

Studies utilizing genetic and pharmacological manipulations in rodent models and neuronal cultures have revealed myriad roles of brain-derived neurotrophic factor (BDNF). Currently, this knowledge of BDNF function is being translated into improvement strategies for several debilitating neurological disorders in which BDNF abnormalities play a prominent role. Common among the BDNF-related disorders are irregular trafficking and release of mature BDNF (mBDNF) and/or its prodomain predecessor, proBDNF. Thus, investigating the conditions required for proper trafficking and release of BDNF is an essential step toward understanding and potentially improving these neurological disorders. This paper will provide examples of disorders related to BDNF release and serve as a review of the techniques being used to study the trafficking and release of BDNF.

## 1. Introduction

Molecular, electrical, and structural properties of neurons are all regulated in part by the most widely expressed neurotrophin in the mammalian central nervous system (CNS), brain-derived neurotrophic factor (BDNF). Mature BDNF (mBDNF) binds with high affinity to the tyrosine kinase receptor, TrkB, and most of the effects of BDNF are due to the intracellular cascades resulting from BDNF-TrkB signaling. The prodomain predecessor of BDNF, proBDNF, is also secreted, but preferentially binds to the pan-neurotrophin receptor p75NTR, triggering intracellular cascades with very different effects than those elicited by BDNF-TrkB signaling [1, 2].

BDNF and proBDNF play essential roles in neuronal development, plasticity, differentiation, and survival (see [3, 4] for reviews). With such widespread function, there is a substantial possibility for BDNF malfunction. In Alzheimer's disease patients, BDNF mRNA was reduced 50% in nuclei of the basal forebrain, the origin of the cholinergic neurons that innervate the cortex and hippocampus, and a region associated with the cognitive deterioration seen in Alzheimer's [5]. Neuronal survival is compromised in Huntington's disease as a result of an attenuated TrkB-Shc signaling pathway [6]. Thus, many neurological disorders have been associated with BDNF, with abnormalities occurring anywhere from the transcription of the *BDNF *gene to the receptor-mediated signaling cascades.

Between the transcription and signaling of BDNF are the equally important trafficking and release of it. BDNF in its pro- and/or mature form is packaged in the trans-Golgi network (TGN) and will see one of two possible fates: storage in dense-core granules for release via activity-regulated secretory pathways, or smaller BDNF vesicles can bud off from the TGN for release through a constitutive pathway (reviewed in [7]). Mice with a genetic knockout (KO) of carboxypeptidase E, a transmembrane receptor in the TGN that directs proBDNF to the secretory pathway, exhibited a wide range of problems in dendritic spine morphology, accompanied by behavioral complications related to learning and memory [8]. A recent study found very different patterns of intracellular cascades, yielding large observable differences in spine morphology, resulting from acute or gradual exogenous BDNF application, even when final BDNF concentration was held constant [9]. These results demonstrate that not only are concentrations of BDNF important for neuronal function, but so are the ways in which BDNF is released—either constitutively (analogous to gradual and chronic application) or in an activity-dependent manner (analogous to rapid and acute application).

Within recent years, knowledge of BDNF trafficking and release, and the techniques used to study them, has improved dramatically [10–13]. With these advancements, research is now able to shift from investigation of normal BDNF dynamics to the study of abnormal BDNF dynamics as they pertain to particular disorders. However, only some such investigations have been conducted [14–16]. In this paper, three tools currently being used to study BDNF trafficking and/or release are reviewed: BDNF ELISA, BDNF tagged with enhanced green fluorescent protein from the jellyfish* Aequorea victoria *(BDNF-eGFP), and BDNF-pHluorin, which is tagged with the superecliptic variant of pHluorin, a pH-sensitive mutant of eGFP (see Table 1). Discussion will then shift towards the value of these methods in studying the role of BDNF in the following neurological disorders: (i) Val66met, a subclinical condition defined by a common single nucleotide polymorphism in (SNP) in the pro region of BDNF. It is associated with memory complications and decreased hippocampal and cortical volume [17]; (ii) neurodevelopmental disorders such as Rett syndrome and Fragile X syndrome; (iii) Huntington's disease, an inherited neurodegenerative disorder.

## 2. BDNF ELISA

Enzyme-linked immunosorbent assay (ELISA) involve linking a protein-specific antibody to an enzyme (e.g., horseradish peroxidase), which then processes common conjugated substrates to yield an absorbance reading that is proportional to the amount of antibody bound to the antigen being studied. Standard curves using known concentrations of antigen are made so that the absorbance can be used to ascertain the unknown concentration of the antigen. The sandwich variation of ELISA is most commonly used to study BDNF levels. Recently, a product has been designed to specifically detect proBDNF via ELISA (Adipobioscience), but these do not appear to have been widely used as of yet; most studies have utilized standard BDNF ELISA, which is unable to discriminate between mBDNF and proBDNF.

Prior to BDNF ELISA, BDNF mRNA levels served as the most common measure of cellular BDNF content. It was clear using RT-PCR or *in situ *hybridization techniques that BDNF transcripts increase in an activity-dependent manner [18], but the amount of BDNF protein being produced could not be quantified because of very small endogenous concentrations [19]. The first experiment to use BDNF ELISA definitively showed discrepancies between BDNF mRNA and protein levels; there were numerous temporal and spatial differences in the dynamics of the mRNA and protein, suggesting involvement of posttranslational mechanisms that rendered BDNF mRNA an incomplete measure of the presence of BDNF [19]. There are now commerciallyavailable kits for BDNF ELISA (Promega's BDNF Emax ImmunoAssay System) that have become a staple in studies comparing BDNF levels across different brain regions and experimental groups [20]. Additionally, a variant named BDNF ELISA *in situ *has been shown to be much more accurate in detecting release of endogenous BDNF in primary neuronal cultures, making it possible to detect the minute changes in extracellular BDNF that occur upon electrical stimulation [13].

With BDNF ELISA, temporal and spatial information cannot be attained—only the amount of BDNF present or released in a sample throughout a protracted time span can be determined [21]. The major advantages in using ELISA are genetic manipulation is not necessary; blood serum levels of BDNF are reflective of brain BDNF levels [22] and can be readily measured in human subjects using BDNF-ELISA [23]; endogenous BDNF release and/or levels can be detected at a sensitivity of 1–3 pg/mL [19, 24]; and BDNF can be measured without destroying the tissue being studied—perfused liquids can be measured for their BDNF content [13, 25]. These last two points are particularly important in that they offer BDNF ELISA an advantage over Western immunoblotting. However, Western immunoblots can be more specific than ELISA in distinguishing BDNF and proBDNF [26], and in elucidating the cascades involved in BDNF signaling effects [9].

## 3. BDNF-eGFP

Several types of mammalian proteins have been tagged with wildtype green fluorescent protein (GFP) from the jellyfish *Aequorea victoria *and, given that the subcellular localization of the fusion proteins are intact, these tagged proteins allow the study of their trafficking mechanisms (reviewed in [27]). The first BDNF-eGFP fusion protein was generated by inserting the rat preproBDNF gene into a CMV promoter-driven vector expressing eGFP using DNA recombination [28]. The primary localization of BDNF-eGFP to somatodendritic compartments matched previous immunocytochemical findings, showing that BDNF-eGFP was trafficked identically to the endogenous BDNF protein [28]. Importantly, the synthesis and posttranslational processing of BDNF-GFP were also reported to be largely the same as endogenous BDNF [11, 28]. However, a recent study showed that the ratio of proBDNF-eGFP/mBDNF-eGFP is higher than the endogenous proBDNF/mBDNF ratio [29].

BDNF-eGFP offered the ability to image dynamic activity of BDNF, enabling the discovery that BDNF travels trans-synaptically in an activity-dependent manner, providing great support that BDNF is involved in synaptic plasticity [11]. Anterograde transport and release from axon terminals [11], autocrine and paracrine dendritic BDNF release [12], and the need for back-propagating action potentials in dendritic release [30] are among other important findings that were only possible with time-lapse BDNF-eGFP imaging in live neurons. However, higher than endogenous levels of BDNF expression have been necessary thus far to aid in the visualization of BDNF-eGFP [28, 31].

A unique advantage offered by fluorescent tagging is the ability to observe BDNF trafficking, which involves packaging, transport, and localization of BDNF. With trafficking abnormalities implicated in Alzheimer's disease [32], Huntington's disease [33], and others (reviewed in [34]), the ability to image BDNF with temporal and spatial resolution is critical. Issues with BDNF-eGFP include overexpression of BDNF, inability to distinguish mBDNF and proBDNF, transfection is often inefficient and/or harsh, and there is a requirement for *in vitro *preparation.

**Figure 1:**
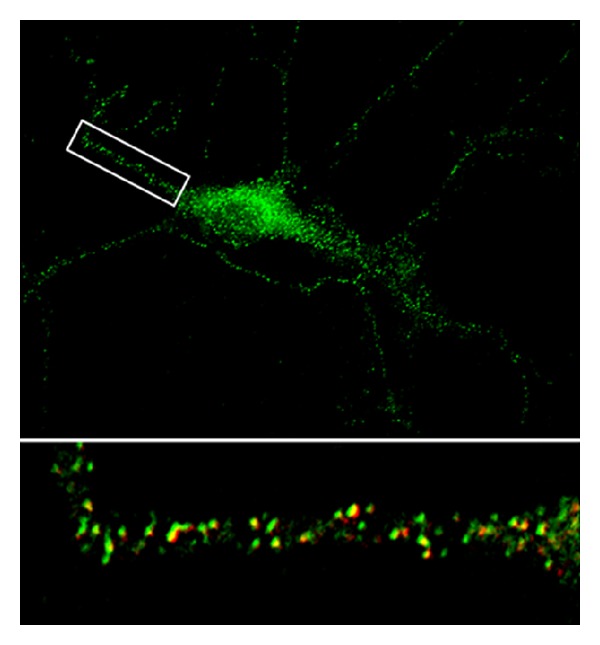
A pyramidal neuron expressing BDNF-eGFP. Insert shows BDNF-eGFP puncta in dendrites, which colocalize with the secretory granule marker SG2 (red).

Figure 1 shows an example of a hippocampal neuron in primary culture after transfection with BDNF-eGFP, with subsequent immunolabeling with SGA2, a marker of secretory granules. This image shows that BDNF-eGFP is trafficked into SGA2-positive secretory granules, evidence that it is processed similarly to endogenous BDNF. We are currently using this approach for time-lapse imaging of BDNF trafficking in neurons obtained from *Mecp2*-deficient mice, a model of Rett syndrome (see below).

Several methods for introduction of the BDNF-eGFP construct are available, including liposome-based reagents (e.g., Lipofectamine) and viral transductions. Viral transduction may offer a gentler method of inducing the expression of BDNF-eGFP in cells. The landmark study by Egan et al. [35] transduced BDNF-eGFP plasmids into hippocampal cultures using a Sindbis virus in their discovery that val-BDNF is trafficked differently than met-BDNF (discussed below, [35]). Several studies have since used Sindbis virus-mediated transduction to investigate trafficking and release of variants of BDNF [36]. One mouse model has been developed in which the human BDNF (hBDNF) gene with a C-terminal eGFP gene was integrated into the mouse genome [37]. The hBDNF-eGFP gene, particularly its exons I and IV, is upregulated in response to neuronal activity, recapitulating the behavior of wildtype mouse BDNF [38]. However, hBDNF-eGFP in this mouse line is expressed in addition to the endogenous untagged BDNF, which leads to BDNF overexpression with unknown consequences [38]. A knock-in mouse model in which the endogenous BDNF is replaced by eGFP-tagged BDNF would be a way to circumvent the issue of BDNF overexpression that is prevalent in BDNF-eGFP research. Because of the incredible complexity of the rodent BDNF gene, a BDNF-eGFP knock-in mouse has been difficult to generate. However, knock-in mice have been generated that replace wildtype BDNF with the val66met BDNF polymorphism [39]. Thus, a knock-in BDNF-eGFP mouse is plausible and would be an important advancement in BDNF research, as it could even open the possibility for live *in vivo* imaging of BDNF at least in the cerebral and cerebellar cortices, which are accessible to multiphoton excitation microscopy through thinned skulls or cranial windows.

## 4. BDNF-pHluorin

Because of a vesicular proton ATPase, the lumen of secretory granules (including neurotransmitter-containing vesicles) is typically at pH ~ 5.5, meaning that a protein with pH sensitivity in this range can be used as an optical indicator of vesicular release. Genetic mutagenesis of key amino acids that surround the chromophore in wildtype eGFP-generated pHluorin, a mutant with its fluorescence intensity highly variable in the 5.5–7.4 pH range [83], taking advantage of natural variations in fluorescence that occur in the four different protonation states of the GFP chromophore [84]. Synaptobrevin, a membrane protein involved in vesicular release, was tagged on its luminal portion with pHluorin [83]. Because pHluorin fluorescence increases upon exposure to the extracellular environment, it was clear when and where synaptobrevin-containing vesicles fused [83], illustrating how tagging proteins with pHluorin can provide information that standard eGFP tagging cannot. However, when pHluorin-containing vesicles are quenched, they hardly fluoresce, meaning that pHluorin cannot be used in monitoring vesicle trafficking. An additional concern is that, if pHluorin-tagged proteins diffuse out of a vesicle (i.e., cargoes like BDNF), there is a net fluorescence decrease because the local concentration of pHluorin molecules decreases sharply when vesicle fusion is sustained [10, 85]. Similar to BDNF-eGFP, experimental concerns for BDNF-pHluorin include overexpression of BDNF and potentially harsh transfection methods; so far, viral transduction has not been used for BDNF-pHluorin.

**Figure 2:**
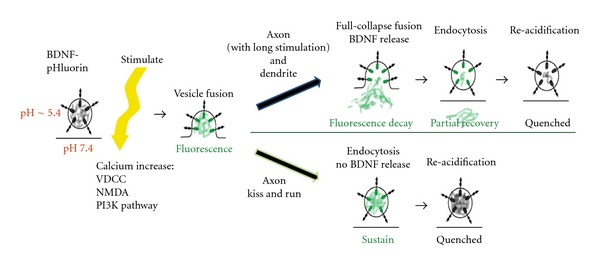
Schematic illustration of how BDNF-pHluorin fluoresces, both inside the vesicle and upon axonal and dendritic vesicular fusion. Before stimulation, BDNF-pHluorin protein shows little fluorescence. If intracellular calcium increase occurs upon electrical stimulation, then BDNF-pHluorin vesicle may fuse, opening up to the pH 7.4 extracellular space, causing a transient spike in fluorescence that can be detected by TIRF microscopy. Different styles of fusion between axon and dendrite are shown, as explained in [10] and in the text. After sustained vesicular fusion as occurs in dendrites, fluorescence will decrease as a result of BDNF-pHluorin diffusion out of vesicles. Kiss-and-run fusion as occurs in stimulated axons will rather show an increase in fluorescence because minimal pHluorin diffuses out of the vesicle. The sticks represent synaptotagmin-4; see [85] for details. Modified from Figure 4(e) in [85].

BDNF-pHluorin was first used in the discovery that synaptotagmin-4 (syt4) regulates exocytosis of BDNF secretory vesicles [85]. This chimeric protein was also used to characterize BDNF release in response to different types of stimulation; theta burst stimulation (TBS) and other LTP-inducing stimulations showed the highest amounts of BDNF release [10]. These two papers comprise the entire body of published BDNF-pHluorin research. Both studies show that BDNF-pHluorin fluorescence increases transiently following neuronal depolarization but only in axonal puncta, meaning that BDNF vesicle fusion proceeds though a process of several repeated fusions, followed by re-acidification and requenching of BDNF-pHluorin, that is, kiss-and-run (Figure 2). On the other hand, the intensity of BDNF-pHluorin puncta in dendrites always showed a net decrease following depolarizing stimulation, indicating full vesicular fusion and BDNF discharge (Figure 2). However, fluorescence increases in the initial fusion events could be detected in both axonal and dendritic puncta using total internal reflectance fluorescence (TIRF) microscopy and faster image acquisition rates [10], enabling detection of many types of single vesicle fusion events. Thus, these studies illustrate how BDNF-pHluorin can be very useful in determining vesicular release kinetics of BDNF. It would be of great interest to investigate if BDNF vesicular release kinetics is altered in BDNF-related disorders, but none such studies have been conducted to date.

**Figure 3:**
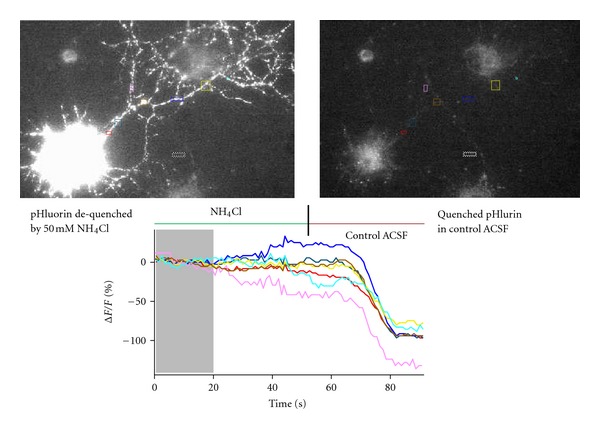
Left panel shows a BDNF-pH expressing hippocampal neuron in the presence of 50 mM NH_4_Cl. Right panel shows the same cell 80 sec after standard ACSF solution was applied. The graph shows the time course of the fractional change of BDNF-pH intensity (background-subtracted delta F/F_0_) from pixels within the color-coded regions of interest (ROIs) shown in the panels above. The change from NH_4_Cl-containing ACSF to control buffer quenches BDNF-pH within acidic secretory granules. Time-lapse was performed in an inverted microscope with a Hg-lamp and a cooled CCD camera. Neurons were imaged with a 60x 1.45 NA oil-immersion objective, exposure times ranged from 50–100 ms, and images were taken at 1 frame per second (fps).

**Figure 4:**
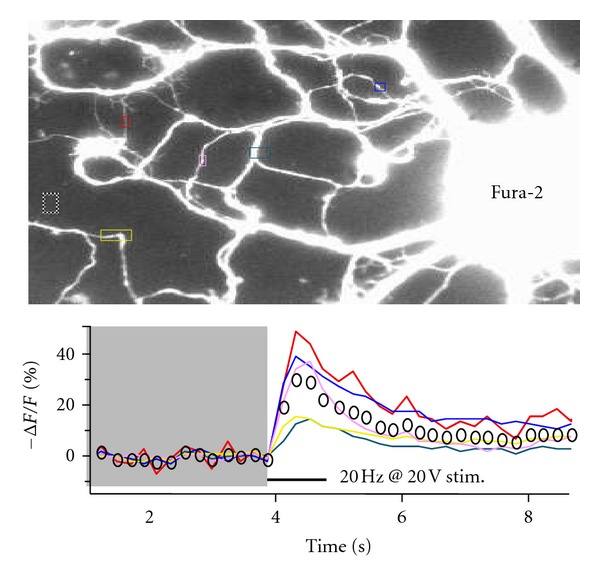
To confirm the viability of neurons transfected with BDNF-pH and their responsiveness to extracellular field stimulation, Ca^2+^ imaging was performed in neurons labeled with fura-2 AM. Upon electrical stimulation through field Pt wires, Ca^2+^ transiently increased in several dendrites. The graph shows the time course of background-subtracted delta F/F_0_ of 380 nm-excited fura-2 fluorescence intensity (510 nm emission) from the colored ROIs shown in the image. Images taken at 4 fps.

**Figure 5:**
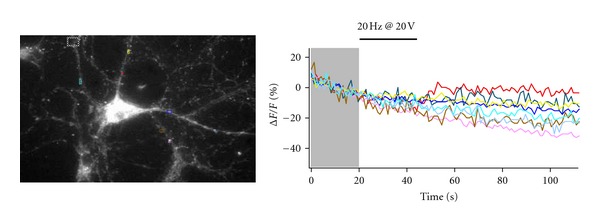
Neuron expressing BDNF-pHluorin and loaded with the Ca^2+^ indicator fura-2 AM (image is of 488 nm-excited BDNF-pHluorin). This cell was sensitive to pH (as in Figure 4) and responded to electrical stimulation with Ca^2+^ transients (using 380 nm excitation, as in Figure 5). Consistent with activity-dependent BDNF release, dendritic BDNF-pHluorin puncta show decreases in intensity (~20% deltaF/F) as a result of BDNF-pHluorin discharge from vesicles following full fusion.

Figures 3 and 4 show unpublished work from our laboratory using a BDNF-pHluorin plasmid from the Poo laboratory [10]; same results were obtained with a BDNF-pHluorin plasmid from the Chapman laboratory [85]. BDNF-pHluorin in cultured hippocampal neurons is sensitive to pH, as demonstrated by its quenching when switching from artificial cerebrospinal fluid (ACSF) containing 50 mM ammonium chloride (NH_4_Cl) to control ACSF (Figure 3). To confirm the viability of neurons transfected with BDNF-pHluorin and their responsiveness to extracellular field stimulation, Ca^2+^ imaging was performed in neurons labeled with the fluorescent dye fura-2AM. Upon stimulation with a 1 sec-20 Hz train at 20 V delivered by Pt wires, Ca^2+^ transiently increased in several dendrites (Figure 4). Using the same stimulation intensity but for longer duration, dendritic BDNF-pHluorin puncta showed activity-dependent destaining consistent with full fusion and BDNF discharge from secretory granules (Figure 5). Current studies in our laboratory use BDNF-pHluorin to study activity-dependent BDNF release in neurons from a mouse model of Rett syndrome.

So far, all studies of BDNF release agree upon the requirement for intracellular Ca^2+^ elevations, and the varying effects of different stimulation frequencies on BDNF release, with TBS and other LTP-inducing protocols eliciting the greatest amounts of BDNF secretion [10, 12, 13, 21]. None of the methods described in this review can provide measures of the effects of BDNF-receptor signaling. Standard Western immunoblotting, and newly developed immunofluorescence assays that do not require tissue homogenization [27] can specifically detect qualitative and quantitative molecular responses to BDNF, complementing the methods reviewed here.

## 5. Application to Neurogenetic Disorders

### 5.1. Val66Met BDNF Polymorphism

An estimated 30–50% of people worldwide are homozygous or heterozygous for a single nucleotide polymorphism (SNP) that has only been observed in the human *BDNF *gene [40]. With this SNP, valine is changed to methionine in the 66th residue of the pro region of proBDNF. This mutation has not been shown to affect expression or signaling of mature BDNF, but improper sorting of met-BDNF-GFP from the trans-Golgi network into secretory vesicles dramatically reduced the amount of vesicles in dendritic compartments, leaving much of the met-BDNF-GFP in the perinuclear region rather than in synaptic regions [35, 41]. Using BDNF ELISA with chronic KCl depolarization, it was very evident that the activity-dependent release of met-BDNF was substantially less, but constitutive release of val-BDNF and met-BDNF was nearly identical [35]. Coimmunoprecipitation showed that the protein sortilin binds to the pro region of BDNF and directs it from the trans-Golgi network into the regulated secretory pathway; the association with sortilin was stronger in val-BDNF than it was in met-BDNF, explaining the trafficking defects observed with met-BDNF [14].

Behavioral and fMRI studies of the consequences of the Val66Met BDNF polymorphism were the first to link human behavior and brain morphology with BDNF action [40]. Subjects heterozygous for met-BDNF scored significantly lower on episodic memory tasks, but not a factual recall task [35]. These results emphasize the importance of regulated BDNF release in memory, particularly in the hippocampus, where BDNF mRNA expression is highest [42], and where it promotes the survival and differentiation of newborn neurons [43]. Additional influences of the BDNF polymorphism were seen in fMRI studies, which revealed that hippocampal activation patterns were abnormal during a memory task [35], and hippocampal and dorsolateral prefrontal cortex volumes were ~10% less in met-BDNF carriers [17].

The results from these behavioral and imaging studies illustrate a range of macroscopic effects that can be predicted by studies of molecular activity: less overall BDNF is being released [35] and several lines of evidence suggest that cleavage of met-proBDNF into mature BDNF is impaired compared to val-proBDNF, meaning that LTP and dendritic arborization will be further impaired because of reduced TrkB activation. In colocalization studies using BDNF-YFP (yellow fluorescent protein), it was recently shown that an association between sortilin and proBDNF at the TGN facilitates cleavage of proBDNF by the protease furin [44]. Because met-proBDNF binds less strongly to sortilin than val-proBDNF, it is highly plausible that cleavage of met-proBDNF is less efficient than cleavage of val-proBDNF. Additionally, proBDNF-GFP was shown to be copackaged into dense-core granules of the activity-dependent secretory pathway with tissue plasminogen activator (tPA) and plasminogen—two proteins that interact to make plasmin [45, 46]. This means that met-proBDNF is no longer colocalized to secretory granules with its primary extracellular protease, so that proBDNF may predominate over mBDNF in the extracellular space. Recent evidence corroborates this idea—the extracellular ratio of mBDNF:proBDNF dramatically increased under depolarizing conditions [29]; without proper activity-dependent release, this ratio maybe reduced, and less mBDNF-TrkB signaling may help to explain the deficits in memory and hippocampal volume seen in Met-BDNF carriers. This latter study illustrates how BDNF-eGFP can be useful in methods besides fluorescence microscopy: anti-GFP antibodies were used to pull down BDNF-eGFP and proBDNF-eGFP in cellular media, and western blotting then quantified relative amounts of BDNF-eGFP and proBDNF-eGFP to show that mBDNF is the dominant species released following high-frequency stimulation [29]. The extracellular mBDNF:proBDNF ratio has yet to be directly studied in Val66Met. This would be an important aspect of the disorder to study, given that extracellular cleavage of proBDNF is essential for LTP, and proBDNF preferentially binds the p75 receptor, which has pro-apoptotic cascades [45, 47].

### 5.2. Neurodevelopmental Disorders Associated with Intellectual Disabilities

There is a well-studied link between the impairment of activity-dependent refinement of synapses and the neurodevelopmental disorder Rett syndrome (RTT), a debilitating disorder that affects ~1 : 15,000 females worldwide (reviewed in [34]). Rett syndrome first manifests itself 6–18 months after birth, with common symptoms being difficulty breathing and deterioration of acquired motor, language, and social skills [48]. Origins of the disorder have been confirmed to be mutations that yield loss of function in *MECP2*, a gene on the X chromosome that encodes for the transcriptional regulator protein MeCP2. MeCP2 binds to A/T-rich sites near methylated CpG islands and can recruit transcriptional repressor and/or activator proteins that can then modify chromatin (reviewed in [49]). Interestingly, one of the many genes regulated by MeCP2 is the *Bdnf *gene. *BDNF* mRNA levels are lower in autopsy brain samples from RTT individuals [50] and BDNF protein levels are decreased in *Mecp2*-deficient mouse models, with more severe decreases in BDNF are associated to more severe RTT-like symptoms [51]. The mechanisms underlying MeCP2 control of BDNF have long been debated, but recent evidence shows that MeCP2 acts to repress transcription of multiple microRNAs that bind to the 3′UTR region of BDNF mRNA and subsequently reduce BDNF protein levels, as measured by ELISA [52]. This finding may explain how loss of MeCP2 function leads to impaired BDNF expression.

The influence of BDNF on RTT is widespread. Autopsies showed that, in hippocampus [53] and neocortex [54] of RTT patients, there was a pronounced decrease in dendritic growth and spine density (but see [55]). This is possibly explained by the reduced expression and phosphorylation of microtubule-associated protein 2 [54], processes which are both modulated by BDNF [56]. Increased expression of BDNF, measured by Western blots [57], BDNF-GFP [31], or ELISA [58], caused improvements in synaptic function, dendrite length, and respiratory function in Rett syndrome mouse models. RTT patients with the Val66Met BDNF polymorphism showed more severe symptoms and an increase in the risk of seizure onset, suggesting that BDNF trafficking and secretion are altered in RTT, not just BDNF expression levels [59]. This idea gains credence with ELISA *in situ *evidence that the total amount of activity-dependent BDNF release is equal to wildtype levels in the *Mecp2*-/y mouse model, even when BDNF expression was less than half wildtype levels and constitutive secretion was less than wildtype levels [16]. Thus, a larger readily releasable pool (RRP) of BDNF in RTT was suggested, but BDNF ELISA cannot confirm this; BDNF-eGFP should be used to follow up this investigation because it can reveal the relative quantities of BDNF vesicles that localize to neurites and therefore can help to uncover an altered RRP of BDNF vesicles in RTT.

Dendritic spine morphology is regulated by activity-dependent BDNF release and synthesis [60]. Abnormal spine morphologies have long been associated with genetic neurodevelopmental disorders, particularly RTT and Fragile X syndrome (FXS) [61, 62]. Postmortem studies of RTT patients showed reduced dendritic spine density in the hippocampus, with a greater ratio of thin/mature spines than in wildtype hippocampus [63]. To aid in future investigations, induced pluripotent stem cells (iPSCs) were recently generated from RTT patient fibroblasts and were differentiated into neurons that recapitulated many characteristics of neurons in RTT patients, namely, reduced spine density [63]. Mature neurons and proliferative neural precursor cells were generated in the process, opening the exciting possibility of sustaining cell lines that recapitulate the RTT phenotype [63]. iPSCs derived from patients afflicted with a neurogenetic disorder open an exciting new avenue in neuroscience research, which thus far has been seldom utilized in studying the involvement of BDNF in neuropathologies.

In Fragile X syndrome, an inherited autism-spectrum disorder associated with intellectual disability that affects 1 : 4000 males and half as many females, mutations in the *FMR1* gene cause a functional absence of Fragile X mental retardation protein (FMRP), a protein that reduces translation by recruiting 4E-BP proteins to the 5′ end of mRNAs [64, 65]. Importantly, FMRP can also work to increase translation by transporting mRNA, such as Rab3a and BDNF mRNA to neurites [65, 66]. Indeed, levels of Rab3a protein, which is important in activity-dependent dense core vesicle docking and fusion at the pre-synaptic terminal, were substantially reduced in tissues from *Fmr1* knockout mice, causing dysfunctional release of neuropeptide vesicles [65]. This finding might provide an explanation as to why hippocampal neurons from *Fmr1* knockout mice show impaired LTP that can be restored by application of recombinant BDNF, even though levels of proBDNF and mBDNF were not significantly different between wildtype and *Fmr1* knockout mice [67], and BDNF was in fact increased in the hippocampus of *Fmr1* knockout mice in another study, as measured by BDNF ELISA [66]. Considering that the levels of BDNF are not negatively affected by loss of FMRP function, but dense core vesicle docking is affected, it is very plausible that BDNF vesicle docking and fusion is affected in FXS, contributing to the observed deficits in LTP and learning [68]. Additionally, one study has linked BDNF trafficking with FXS symptoms, showing an increased propensity for epilepsy in FXS-afflicted men who have the improperly sorted met-BDNF allele [69]. Future studies utilizing BDNF-eGFP or BDNF-pHluorin in neurons from FXS models could elucidate the ways in which BDNF trafficking and vesicle fusion are affected in FXS, providing clues as to why LTP and dendritic spine maturation are impaired in FXS.

There is also evidence that BDNF is upstream of FMRP activity: BDNF, but not neurotrophin-3 application, and overexpression of TrkB was shown to slightly reduce the expression of the *Fmr1 *gene, implicating BDNF-TrkB signaling in the negative regulation of FMRP levels [70]; BDNF also caused dissociation of the FMRP translational repression complex, allowing translation to occur specifically in dendritic compartments [64]. The impact of abnormal communication between BDNF and FMRP that is expected to occur in FXS has not been extensively studied (see [66]), and deserves further investigation.

### 5.3. Huntington's Disease

In contrast to the neuropathologies discussed thus far, Huntington's disease (HD) is a neurodegenerative disorder with symptoms usually appearing in middle age [71]. Its most characteristic symptoms are related to loss of motor function as a result of basal ganglia degeneration, but often before motor symptoms become manifest there are cognitive deficits in verbal learning and working memory, tasks associated with hippocampal and prefrontal areas [72]. HD is a rare inherited disorder that affects 5–10 in 100,000 Caucasians, with fewer reported cases in Japanese populations [73]. HD is associated with 36 or greater CAG repeats in the *huntingtin *gene, producing an abnormal huntingtin protein (htt) with an extended tract of glutamine that is encoded by the CAG repeats. The severity of the disease is proportional to the number of CAG repeats, as the polyglutamine tail causes self-association of htt mutants that can lead to htt aggregates [73]. Unlike those seen in Alzheimer's disease, these mutant htt aggregates do not have intrinsic toxicity, but vesicle trafficking and endocytic recycling are severely impaired in mutant htt, leading to dendritic spine degeneration and eventual cell death ([74, 75], but see [76]).

BDNF-eGFP vesicles colocalized with wildtype and mutant htt, but only wildtype htt was shown to increase velocity and efficiency of BDNF transport to synapses. This effect appeared specific for BDNF vesicles, as mitochondrial transport was unaffected [33]. htt-mediated axonal transport of BDNF requires Huntington-associated protein 1 (HAP1); HAP1 associates with the pro domain of BDNF-eGFP, and this association is reduced with mutant htt and with met-BDNF [15], likely because of impaired complexation with sortilin [44]. This finding suggests impaired activity-dependent release and axonal transport, but normal constitutive release, in HD as a result of improper proBDNF-HAP1-htt interactions. The importance of this finding is apparent when considering that the striatum, the region defined by neurodegeneration in HD, contains very low levels of BDNF mRNA [77] but still requires BDNF protein for survival of striatal inhibitory neurons in mouse HD models [20, 78]. This means that the striatum requires anterograde corticostriatal transport of BDNF in order to avoid striatal degeneration [77–79]. Thus, as revealed by BDNF-GFP, the abnormal anterograde trafficking of BDNF by htt and HAP1 is a major component of striatal neuron degeneration in HD.

Transcription of the *Bdnf *gene is also affected in HD. When wildtype htt was overexpressed, BDNF ELISA showed higher levels of BDNF, and the opposite effect was seen with mutant htt. This is a result of wildtype htt releasing the transcriptional repressor REST/NRSF from a 23 bp DNA sequence called RE1/NRSE that was discovered in one particular *Bdnf *exon. Mutant htt was unable to derepress transcription of the *Bdnf *gene, explaining the reduced BDNF levels seen in HD brains (reviewed in [80]). Other studies corroborate these data, finding that cortical levels of BDNF mRNA negatively correlate with the progression of HD in a mouse model [81], and that overexpression of forebrain BDNF results in increased levels of striatal BDNF, and significant reduction in HD phenotype mouse model [26]. With so many facets of HD mediated by BDNF, there is a clear potential for pharmacological applications: upregulation of BDNF by activity-inducing ampakines restored synaptic plasticity and memory in hippocampus of an HD mouse model by stabilizing actin polymerization in dendritic spines, a process severely impaired in HD [82]. BDNF-GFP trafficking studies, and BDNF ELISA studies of protein levels have proven invaluable thus far in elucidating the role of BDNF in HD. However, the quantal release of BDNF in HD has yet to be visualized, an application well suited for BDNF-pHluorin.

## 6. Conclusion

BDNF is a neuropeptide with a diverse array of functions that modulate neuronal function during early brain development and throughout life. The dynamics of its intracellular transport and activity-dependent regulated release are complex and require sophisticated approaches that are currently in development. To start, methods to study BDNF transport and release must be dynamic in order to properly follow its behavior in live cells. Thanks to the development of BDNF-GFP and BDNF-pHluorin, we are beginning to understand the ways in which BDNF contributes to neuronal function in health and disease. Therapies are currently being developed that will aid in restoring BDNF abnormalities to normal, thereby slowing the progression of diseases like Huntington's and Rett syndrome. Future studies can use the tools discussed in this review to test the efficacy of experimental compounds on BDNF transport and release, using the restoration of proper BDNF dynamics as a preclinical endpoint. Hopefully, these studies will yield novel therapies for the wide range of neurogenetic disorders associated with BDNF dysfunction.

## Figures and Tables

**Table 1:** Summary of advantages and disadvantages of using BDNF ELISA, BDNF-eGFP, BDNF-pHluorin.

Method	Primary purpose	Advantages	Disadvantages	References
BDNF ELISA	Quantify levels of BDNF in homogenized tissue, or levels of released BDNF if using ELISA *in situ *	Genetic manipulations are unnecessary. Endogenous BDNF levels can be quantitatively measured with pg sensitivity. proBDNF-specific ELISA kits are available. If using ELISA *in situ, *the amount of released BDNF can be accurately quantified, and tissue need not be destroyed.	Cannot identify sites of BDNF release, and cannot observe trafficking of BDNF vesicles. ELISA is performed over the course of hours, and changes in BDNF cannot be observed in real time. ELISA *in situ* inhibits BDNF-TrkB signaling.	[13, 19, 20, 22, 24, 51, 57, 59, 65]

BDNF-eGFP	Visualize BDNF trafficking and release in real time	BDNF vesicle dynamics can be observed throughout the cell. Sustained vesicle release can be observed by a relative decline in fluorescence. Can be used in conjunction with western blot to determine relative levels of mBDNF and proBDNF. Downstream signaling of BDNF-eGFP release is similar to that of BDNF.	cDNA plasmids for BDNF-eGFP must be introduced by transfection or viral transduction, which can be harsh on cells, and/or can lead to artificial overexpression of BDNF. Cannot quantify absolute levels of released BDNF.	[11, 12, 28–31, 33, 35, 36]

BDNF-pHluorin	Visualize BDNF vesicle release kinetics in real time	The pH sensitivity of pHluorin enables discrimination between sustained and transient vesicular fusion, which is indicative of how much BDNF is diffusing out of each vesicle. Downstream signaling of BDNF-pHluorin release is similar to that of BDNF.	Because fluorescence is quenched at low pH, BDNF-pHluorin is difficult to track while inside of acidified vesicles, making this tool unsuitable for BDNF trafficking studies. The same disadvantages as BDNF-eGFP also apply.	[10, 85]
